# Diversity of Bacterioplankton and Bacteriobenthos from the Veracruz Reef System, Southwestern Gulf of Mexico

**DOI:** 10.3390/microorganisms9030619

**Published:** 2021-03-17

**Authors:** Citlali Rodríguez-Gómez, Lorena María Durán-Riveroll, Yuri B. Okolodkov, Rosa María Oliart-Ros, Andrea M. García-Casillas, Allan D. Cembella

**Affiliations:** 1 Unidad de Investigación y Desarrollo en Alimentos, Tecnológico Nacional de México/Instituto Tecnológico de Veracruz, Veracruz 91897, Mexico; cileya@yahoo.com (C.R.-G.); rosa.or@veracruz.tecnm.mx (R.M.O.-R.); 2 CONACYT—Departamento de Biotecnología Marina, Centro de Investigación Científica y de Educación Superior de Ensenada, Carretera Tijuana-Ensenada 3918, Ensenada 22860, Baja California, Mexico; 3 Alfred-Wegener-Institut, Helmholtz Zentrum für Polar-und Meeresforschung, 27570 Bremerhaven, Germany; 4 Instituto de Ciencias Marinas y Pesquerías, Universidad Veracruzana, Mar Mediterráneo 314, Fracc. Costa Verde, Boca del Río 94294, Veracruz, Mexico; yuriokolodkov@yahoo.com; 5 Facultad de Ciencias, Universidad Nacional Autónoma de México, Coyoacán, Mexico City 0451, Mexico; garciacasillas@ciencias.unam.mx

**Keywords:** bacterial biodiversity, bacteriobenthos, bacterioplankton, coral reef, Gulf of Mexico, marine bacteria, coastal zone

## Abstract

Bacterial diversity was explored among field samples and cultured isolates from coral reefs within the Veracruz Reef System. Bacterioplankton and bacteriobenthos were characterized by pyrosequencing 16S rRNA genes. Identified sequences belonged to the kingdom Bacteria and classified into 33 phyla. Proteobacteria (likely SAR11 clade) dominated in collective field samples, whereas Firmicutes were the most abundant taxa among cultured isolates. Bioinformatic sorting of sequences to family level revealed 223 bacterial families. Pseudomonadaceae, Exiguobacteraceae and Bacillaceae were dominant among cultured isolates. Vibrionaceae, Alteromonadaceae, and Flavobacteriaceae dominated in reef-associated sediments, whereas Rickettsiaceae and Synechoccaceae were more highly represented in the water column. Bacterial communities from sediments were more diverse than from the water column. This study reveals cryptic bacterial diversity among microenvironmental components of marine microbial reef communities subject to differential influence of anthropogenic stressors. Such investigations are critical for constructing scenarios of environmentally induced shifts in bacterial biodiversity and species composition.

## 1. Introduction

Coral reefs are among the best known examples of ecosystems subject to rapid loss of biodiversity within the last several decades [1]. As arguably the most diverse and complex marine ecosystems, they serve as repositories for genetic richness within their respective communities and as refuges for marine biodiversity. Microbial populations inhabiting coral reef systems differ widely in taxonomic composition, and the microalgal component and particulate organic matter in surrounding waters are also highly diverse [2,3]. Hence, this diversity has a direct but differential influence on host and community health and metabolic processes—the holobiont concept.

One of the main goals in biodiversity research on microbes has been to understand their functional role and impact within and among ecosystems at local, regional, and global scales. Both prokaryotic and eukaryotic microbes play a critical role in primary production and in biogeochemical cycles in the ocean [4], including coral reef ecosystems. Microbial communities inhabit a variety of niches within and surrounding coral reefs; the significance of this diversity can be more readily interpreted by first describing the microbiome patterns. As a first step, taxonomic and phylogenetic assessments are needed components of the multidisciplinary approach to define the habitat composition under normal ambient and stressed conditions. Nevertheless, this quest has only recently become feasible and affordable with ecogenomic approaches by application of high throughput sequencing technologies for direct sequencing of polymerase chain reaction (PCR) products from environmental samples. Among alternative technologies, next-generation sequencing (NGS) has been successfully applied for large-scale biodiversity analysis based upon 16S rRNA genes from bacteria in a variety of habitats [5].

The Veracruz Reef System (VRS) comprises 23 reefs within a marine national park adjacent to the municipalities of Veracruz, Boca del Rio and Alvarado in the southwestern Gulf of Mexico [6]. The VRS is located close to a terrigenous area generating environmental impacts and anthropogenic stressors unsuitable for the flourishing of such a reef ecosystem [7]. Since the founding of the city of Veracruz in 1519, no reef system in Mexico has been subjected to as many intense anthropogenic stressors as the VRS; agricultural and industrial sewage loading from the La Antigua, Jamapa and Papaloapan rivers, urban water discharges, ship groundings, oil spills, port operations and fishing activities are some examples of these stressors [7,8]. Variability of the water temperature and salinity in the VRS is mainly determined by the discharges of the Jamapa and Papaloapan rivers. During the rainy season the Jamapa River has a very high discharge, but, in general, the discharge rate is low during most of the year [9,10]. In comparison, the Papaloapan River has an higher annual discharge rate southeast of the VRS [10].

The multi-source but anthropogenically created stress regime provides a suitable environment for exploring how microbial communities have adapted to these unusual conditions. For some reefs within the VRS the coral coverage has been seriously negatively affected while algal biomass and spatial coverage have been increasing [11]. If even slightly elevated temperature from global ocean warming affects the taxonomic composition of corals and algal-associated bacteria [2], adaptive changes in both the water column and sediment microbial communities would be expected, thereby conserving its “seed bank” status for corals [12]. Some bacteria may confer beneficial effects upon corals by compensating for their lack of an immune system through the production of antibacterial compounds [13], thereby fulfilling the probiotic hypothesis [14].

Conservation of function and ecosystem services are essential to maintaining healthy reef systems, but to date there are no published comparative studies about the microorganisms present in the VRS, and the functional role they could play in maintaining diversity and ecosystem stability. Although the close relationship between coral reefs and the presence of a unique microbiota has been documented elsewhere [15,16], data on the taxonomic and phylogenetic composition of bacterioplankton and bacteriobenthos from the VRS are lacking.

The main objective of this work was to determine if there are discernable bacterial diversity differences between two adjacent coral reefs of the VRS—one close to the port of Veracruz and thus heavily impacted, and the other more distant and less affected by human activities. We analyzed the diversity of bacteria from field populations collected in different environmental compartments—pelagic water column, sediment, and external mucus from corals—from one conserved reef and compared the composition with a more heavily impacted reef to obtain basic knowledge regarding the microbiota. Although we are aware of the culture selection bias, we also compiled DNA sequencing datasets for bacterial diversity from cultured isolates collected from the VRS for comparison. Pyrosequencing of 16S ribosomal DNA was performed from bacterial samples of communities from both reefs to detect even low-abundance members of the microbial population assemblage and to achieve an appropriate degree of taxonomic resolution. A high taxonomic and phylogenetic diversity of marine bacteria was revealed for the first time from various ecological compartments in a coral reef environment in the southern Gulf of Mexico.

## 2. Materials and Methods

### 2.1. Field Sampling and Processing

**Figure 1:**
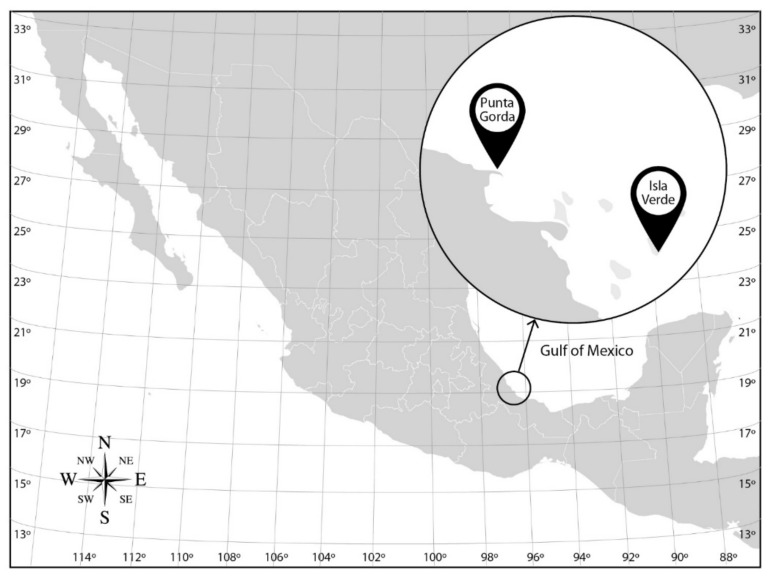
Sampling sites for bacterioplankton and bacteriobenthos from the Veracruz Reef System (VRS) on the Gulf of Mexico coast.

This study focused on two coral reefs of the VRS along the Gulf of Mexico coast: Isla Verde Reef (IVR) (19°11′54.1″ N, 96°04′0.7″ W) located offshore from Veracruz, and Punta Gorda Reef (PGR) (19°14′51.8 N, 96°10′25.2 W) north of the city and more heavily affected by adjacent port activities (Figure 1). Data on several ambient environmental parameters were simultaneously collected with bacterial samples. Conductivity, depth, and temperature were measured in situ with a CTD (Seabird 19 plus Bellevue, WA, USA) and dissolved oxygen with a coupled probe (Oakton RDO, Vernon Hills, IL, USA). Seawater pH was measured later in the laboratory (Corning 440 probe, Salt Lake City, UT, USA). 

The IVR was sampled during the rainy season in August 2009 for bacteria to isolate into culture from the water column (W) (1.5 m depth), sediments (S), seagrass (*Thalassia testudinum* Banks ex König) (T), and from a mucus-like uncharacterized biofilm upon the respective substrate (B). The natural bacterial community was sampled from the water column (11 m depth) and underlying sediments from the same location at IVR in the rainy season in September 2010, and in March 2012 during a transitional period from dominant northerly winds to the dry season. In addition, during the latter sampling period, mucus slurry (5 mL) was carefully collected with sterile syringes from the surface of the massive starlet coral *Siderastrea siderea* Ellis et Solander for comparison of bacterial composition from apparently healthy specimens and from corals afflicted with prominent necrotic black patches. The more anthropogenically impacted PGR system was also sampled from the water column (3.4 m depth) for bacterioplankton and bacteriobenthos from sediments in May 2012 in the dry season.

Seawater samples from the water column were collected in UV-sterilized 20 L bottles, and sediments in UV-sterilized plastic bags. Coral reef mucus was sampled and retained in sterile syringes. All samples were rapidly transported (<2 h) in an ice chest with frozen gel packs to the laboratory. Water column samples were successively filtered with a vacuum pump (Brinkmann Büchi B-169, Flawil, Switzerland) at 20 mbar through 5, 1.2 and 0.22 µm pore size nitrocellulose membranes (47 mm diameter) (Millipore, Billerica, MA, USA). Sediments were first washed with 5 L heat-sterilized seawater (121 °C, 15 min) and the aqueous slurry was filtered as above for the seawater samples. Filters were cut into four equal quadrants for different experiments: one quadrant each of the 5 µm pore size membranes of both water column and sediment samples were used for bacterial cultures, whereas 1.2 and 0.22 µm membranes were retained for rDNA analysis. DNA samples were archived frozen at −70 °C in 0.22 µm sterile-filtered STET lysis solution (8% sucrose, 5% Triton X-100, 50 mM Tris, 50 mM EDTA, pH = 8) (MF Millipore, Merck, Darmstadt, Germany).

### 2.2. Bacterial Culturing

Bacterial cultures from IVR seawater, sediment, seagrass, and mucus biofilm were grown in LB broth (bactotryptone 10 g·L^−1^, NaCl 5 g·L^−1^, yeast extract 5 g·L^−1^) prepared with seawater of the sampling zone (salinity 36) in 250 mL glass bottles placed upon a shaker platform operated at 100 rpm. All cultures exhibiting growth were transferred onto LB (20 g L^−1^) agar plates by streaking with a flame-sterilized bacterial loop. Each isolated colony strain was characterized based on the following criteria: colony characteristics (shape, size, elevation, borders, color, consistency, and transmitted and reflected light), cell shape, Gram-staining and 16S rDNA gene sequence. 

Experiments on isolates in broth culture were performed over a broad temperature range of 1 to 75 °C with intermediate intervals of 33, 40, 60, 65, and 70 °C to determine temperature tolerance and growth characteristics. Generation times were calculated by spectrophotometry (SmartSpec 3000 Biorad, Hercules, CA, USA) from the change in optical density measured at 650 nm. Calculated parameters for growth kinetics were specific growth rate (µ) and duplication time (dt) determined through the culture growth cycle [17], according to the following equation:

µ = ln(*x*_2_/*x*_1_)/(*t*_1_-*t*_2_)

where *x*_2_ and *x*_1_ are the optical density (650 nm) at time *t*_1_ and *t*_2_, respectively.

### 2.3. DNA Extraction and PCR Analysis

Total DNA was extracted and prepared for pyrosequencing for comparison of the bacterial taxonomic composition of diverse ecological compartments from two different coral reefs of the VRS. DNA was recovered from the isolated cultured strains and from the 1.2 and 0.22 µm-filtered natural seawater and sediment samples, and mucus slurry from corals, to construct the DNA library. 

DNA extraction followed well established methods [18,19], with slight modifications as specified here: after overnight incubation (40 mL) broth cultures were centrifuged at 10,000× *g* (Beckman J2–21, Beckman, Pasadena, CA, USA) at 5 °C for 10 min to pellet cells. Pellets were re-suspended in 500 µL STET lysis solution for 15 min, then transferred to a 1.5 mL Eppendorf microfuge tube with 100 µL of lysozyme, mixed by pipetting, and incubated at 37 °C for 45 min. The reaction was stopped at 97 °C by 50 s immersion in a hot water bath. Samples were centrifuged (Model 5415 R, Eppendorf, Hamburg, Germany) at 16,000× *g* for 12 min at 4 °C. The supernatant was pipetted into a new Eppendorf tube and protein was precipitated with 500 µL phenol by gentle mixing. Each sample was centrifuged as above, and the supernatant was transferred to a new 1.5 mL Eppendorf tube. Tubes were placed in ice bath for 5–10 min after adding 50 µL sterile 4M LiCl, then centrifuged at 16,000× *g* for 12 min at 4 °C, after which each supernatant was transferred into a new 1000 µL Eppendorf tube. Samples were precipitated with 500 µL isopropanol at room temperature for 5 min. Samples were centrifuged as described and supernatants were discarded. Then 100 µL of 80% ice-cold ethanol was added and decanted immediately, after which the DNA sample adhered to the vial wall was dried at room temperature for 2 h. Finally, the samples were re-suspended in 50 µL sterile nuclease-free water.

Size and DNA quality were verified by electrophoresis (110 V) in a 1% agarose gel using lambda phage as reference ladder and digested with *Eco*R1 + *Hin*dIII restriction enzymes. DNA was quantified at 260 nm in a spectrophotometer (Agilent 8453, Agilent, Santa Clara, CA, USA) and stored at −20 °C until further analysis.

The PCR was run in a BioRad thermal cycler (MJ Mini/Bio-Rad, Hercules, CA, USA) with bacteria-specific primers 27F and 1492R, as described for coral-associated bacteria [15]. The conditions for PCR were: hold for 2.5 min at 94 °C for the initial denaturation step followed by 30 cycles at 94 °C for 1.5 min, an annealing step at 54 °C for 1.5 min, followed by an extension step at 72 °C for 2 min. The last step lasted 7 min at 72°C. The resulting products (*ca*. 1.5 kb) were purified with a Wizard purification kit (Promega, Madison, WI, USA), and resuspended in heat-sterilized water prepared with a Millipore RiOs-Di 3 water purification system (Merck KGaA, Darmstadt, Germany) to a final concentration of 500 ng µL^−1^. The 16S rRNA gene recovered from each strain was checked for purity with a NanoDrop spectrophotometer (2000C/Bio-Rad, Hercules CA, USA). 

The amplified product was sequenced by the Sanger method (Applied Biosystems Hitachi 3500xL Genetic Analyzer, Foster City, CA, USA) at the Biotechnology Institute, Universidad Nacional Autónoma de México, Cuernavaca, Morelos, Mexico (IBT). Sequences were edited and analyzed with 4Peaks software (Nucleobytes, Aalsmeer, Netherlands) by processing with the 4Peak 1.7.1 program, using the fragments considered in the program-calculated threshold. Identification and comparison of the sequences was conducted by Basic Local Alignment Search Tool (BLAST) and Ribosomal Database Project (RDP) searches. 

### 2.4. Pyrosequencing and DNA Sequence Analysis 

The 16S rRNA was amplified with the universal primer 27F, which included the primer B adaptor for pyrosequencing on the 5’ end. The 1492R included the sequencing primer A and an 8 bp barcode error-correcting Hamming sequence on the 5’ end, slightly modified as previously described [2]. Each DNA sample was amplified by four replicate PCR reactions (then combined) and purified with an Ultra Clean^TM^ Gel Spin^TM^ DNA extraction kit (Mo Bio, Carlsbad, CA, USA). Once purified, the products were quantified by Quant-it PicoGreen assay (Invitrogen, Carlsbad, CA, USA) after pooling the samples at a concentration of 240 ng µL^−1^ in an equimolar amount. For quality control and to search for fragments around 500 bp, the pool was checked with a Bioanalyzer chip (Agilent, Santa Clara, CA, USA) with a high sensitivity kit.

Pyrosequencing (3 µL sample) was performed on a Roche 454 GS Junior (454 Life Sciences, Brandford, CT, USA) sequencing platform following manufacturer’s directions. The resulting sequences were analyzed with free online QIIME software (www.qiime.org, accessed on 3 February 2021), based upon the original bioinformatic scheme of Caporaso et al. [20], but following more recent QUIIME editing protocols for bacterial sequences [21,22]. Edited sequences with chimeras removed were compared by default to the taxonomic data from the Ribosomal Database Project (RDP) for identifying 16S genes [23]. The sequences were sorted to the family level to better characterize the water column and sediment microbiomes. Sequences were clustered into operational taxonomic units (OTUs) based upon >97% similarity criterion and then graphs were generated after statistical analysis with QIIME. Raw high-throughput sequencing reads were deposited in the National Center for Biotechnology Information (NCBI) Sequence Read Archive (SRA) GenBank database (experiment accession numbers MN103868—MN103887; BioProject submission ID numbers: SUB5868169; SUB5868184).

### 2.5. Diversity and Evenness Analysis

Bacterial diversity was calculated using the Shannon-Wiener diversity index [24], according to the equation:

H’ = −∑^R^_i = 1_ pi⋯Ln pi (1)

where *H’* = Shannon-Wiener diversity index; *R* = richness; and *P_i_* = proportion of individuals belonging to the taxonomic level (family).

Evenness, a diversity index that quantifies equality of the community by integrating diversity with taxon richness, was calculated as Pielou’s evenness Index by the following equation [25]:

J’ = H’/H’_max_ (2)

where *J’* = Pielou’s evenness index; *H’* = Shannon-Wiener diversity index; and *H’* = maximum possible value of *H’* if the abundance of the taxa were perfectly equal, calculated as follows:

H’_max_ = Ln⋯R (3)

## 3. Results

The time and site of collection of samples for cultures and sequencing of natural bacterial populations, and relevant environmental data, are given with the sampling codes in Table 1.

Fifteen thermophilic and ten mesophilic isolates were successfully cultivated from field samples from the VRS. Samples and isolates of various compartments are coded as follows: water column (W), seagrass (*Thalassia testudinum*) (T), sediments (S), and mucus biofilm (B). Most thermophilic isolates were Gram-positive [G(+)], whereas the majority of the mesophiles were G(-). In particular, the thermophilic isolates were similar in morphology and physiological characteristics, with four exceptions (isolates IVR-T-12-21, IVR-T-09-31, IVR-B-09-42, and IVR-W-10-2 from Isla Verde reef (IVR) (Table 1), which tended to form biofilms. According to the BLAST search criteria, the *Geobacillus* strains IVR-B-09-42, IVR-B-09-44, and IVR-B-09-45 from IVR were aerobic bacilli, neutrophilic and catalase positive. 

Most thermophilic isolates exhibited bacillary-form with cells ranging from 0.5–3 μm length and about 0.6 μm diameter. Greater morphological variation was found among the mesophilic colonies, which showed both bacillary and coccoid forms of cells in equal proportion. Growth calculated from change in optical density over a broad range of NaCl concentrations (0.5–2 M) indicated halophilic tendencies for all isolates.

Maximum permissive isolation temperatures were 70 and 75 °C from biofilms, and from water and sediment, respectively, at 11 m depth at IVR. Two strains, IVR-W-10-51 from the water column and IVR-S-10-1 from sediment were at first assumed to be psychrophilic because they were isolated at 1 °C but both grew rapidly at 33 °C and generated sufficient biomass for phylogenetic analysis within 15 h. 

### 3.1. Phylogeny of Cultured Isolates 

**Figure 2:**
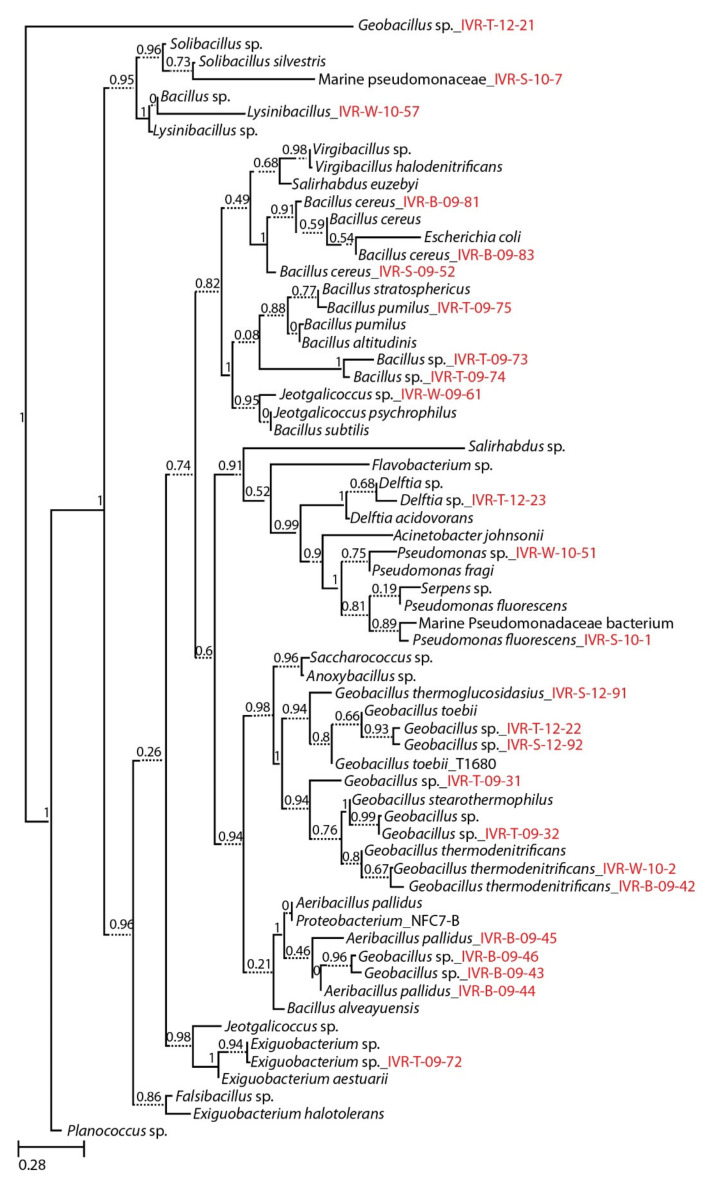
Molecular phylogeny of cultured marine bacterial strains from the Veracruz Reef System constructed by Maximum Likelihood with reference to the 16S rRNA gene sequences from the RDP data base. Numbers at nodes indicate percentages of occurrence in 1000 bootstrapped trees. The scale bar indicates evolutionary distance (0.28 substitutions per site). Strains sequenced in this study are denoted by isolate numbers in red.

Isolates were first classified according to the percentage similarity of the 16S rDNA sequences [26] and then their phylogenetic relationships was confirmed and presented in the phylogenetic tree (Figure 2). Sequenced amplicon results compared with the BLAST and RDP analyses are shown separately for thermophilic (Table 2) and mesophilic (Table 3) cultured isolates from the VRS. Results from both databases were similar for these temperature-defined bacterial groups, but most thermophilic isolates, dominated by Firmicutes in the RDP analysis, were below the expected 97% similarity threshold. This indicates a high likelihood that these taxa are reported here for the first time (Table 2). Nevertheless, there are inherent but mostly minor discrepancies between BLAST and RDP search results that are not easy to define. For example, IVR-B-09-45 shows 100% BLAST similarity with *Aeribacillus pallidus*, but the RDP species similarity does not confirm that the sequences are identical (Table 2).

### 3.2. Taxonomic and Phylogenetic Affinities of Natural Populations of Reef-Associated Bacteria

Analysis within QIIME software yielded 7902 rRNA amplicons from among all water column and sediment samples. The average sequence length was 1128 bp (median = 849), with an average of 640 sequences per sample (median = 277). The number of distinct sequences retrieved varied widely among samples from different compartments between the two reef systems: from IVR-NW-10, 129 sequences and 100 genotypes; IVR-NS-10, 1113 sequences and 277 genotypes; IVR-NW-12, 3962 sequences and 2780 genotypes; IVR-NS-12, 410 sequences and 86 genotypes; PGR-NW-12, 849 sequences and 638 genotypes; and PGR-NS-12, 1320 sequences and 519 genotypes. As expected, comparison of distinct sequences recovered from a combined mix of all bacterial cultured isolates (IVR-CMix) from IVR revealed a highly reduced number (119 sequences and 80 genotypes) relative to the natural population.

All sequences were classified into 33 bacterial phyla (Table 4), whereby Proteobacteria were found in all samples, but with a higher percentage of sequence-referenced taxa represented from IVR (lowest: 56.3%) than from PGR (highest: 45.8%). In comparison, among the cultured isolates, the Firmicutes (65.5%) were more heavily represented than Proteobacteria (32.8%). Bacteroidetes were present in all environmental samples, as a sub-dominant group after Proteobacteria in a sediment sample from PGR (19.4%). Environmental samples from the water column contained Cyanobacteria as the second major phylum (from 21.6 to 25.6%) after Proteobacteria, although they were scarcely represented in sediment samples (2.9–8.6%). Lentisphaerae were present in all sediment samples, but with higher presence at IVR (6.8%) than at PGR (1.7%).

In general, based upon the total number of taxa represented among different major phylogenetic groups, bacterial samples recovered from sediments were more diverse than those from the water column at both reefs. For example, in sediments from PGR, only one phylum (Lentisphaerae) was absent from the total list, but it was present in sediments from IVR. In contrast, the phylum Lentisphaerae was absent from all water column samples.

Among mesophilic isolates, the Firmicutes were again dominant, with γ-Proteobacteria represented by *Pseudomonas* Migula; the main identified genera were *Jeotgalicoccus* Yoon et al.*, Exiguobacterium* Collins et al.*, Bacillus* Ehrenb, and *Pseudomonas* Migula (Table 3). 

The class α-proteobacteria was dominant in the water column at the two reefs (30–40%), and both reefs shared the Synechococcophycideae group within the water column. In the sediments, after α-Proteobacteria, the class γ-Proteobacteria was the second most represented taxonomic group (10–18%) at both reefs. In contrast, according to the pyrosequencing analysis (Table 4), the class Bacilli was the most highly represented among the cultured isolates, comprising more than a half of the retrieved sequences (63.9%).

Alteromonadales was the most abundant order in sediment samples at IVR (11.3–11.7%), but was absent from PGR, where most taxa found at the latter reef belonged to the order Flavobacteriales (16.1%). In the water column, the order Synechococcales was the most abundant (23.3%) at IVR in the transition between high northerly winds and the dry season, whereas Rickettsiales dominated (26%) in the dry season. This latter order was also a major component found at PGR in the dry season, representing 11.3%, along with similar percentages for Rhodobacterales (11.2%) and Lentisphaerales (11.3%). The high percentage of Synechococcales was not found at PGR; this group was absent from PGR and was replaced by Oceanospirillales as co-dominant order (Table 4).

**Figure 3:**
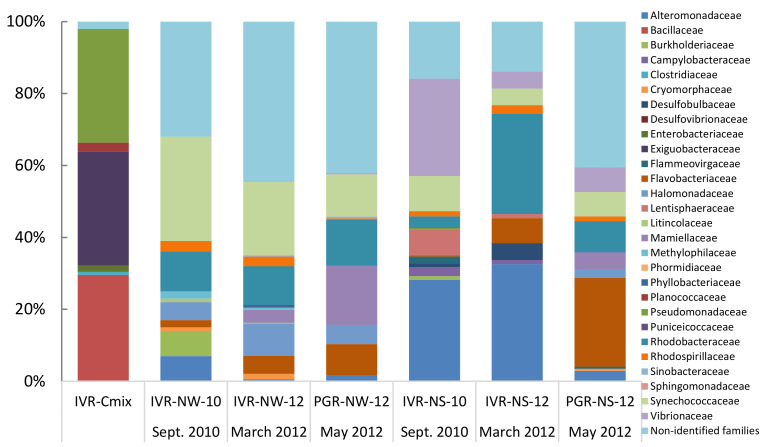
Relative composition (% total) of identified bacterial families represented by 16S rDNA sequences from cultures (IVR-CMix) and environmental samples collected at the Veracruz Reef System from different years (indicated by numbers), seasons and ecological compartments (N = Natural (non-cultured); W = Water column; S = Sediments).

In total, 223 families of bacteria were identified from the two reef systems, with samples from the water column containing lower bacterial family diversity than from sediments. QIIME analysis showed that in general sediments recovered from IVR in September during the rainy season exhibited more taxonomic diversity (maximum 119 families at IVR-NS-10) than from the March transitional period to the dry season from PGR, representing 98 families, and IVR with 52 families (Table 4; Figure 3). Flavobacteriaceae comprised the most abundant taxa recovered from PGR sediments; members of this family were found in all samples, but at lower relative abundance in sediments (0.9–1.7%) than from the water column (2.3–8.8%) at IVR. Taxa from the Synechoccaceae, Flavobacteriaceae, Alteromonadaceae, and Rickettsiales-related families, tended to dominate, and were present in both water and sediment samples, but whereas Rhodobacteraceae and Rhodospirillaceae were also represented in all samples, they were not dominant (typically < 3%) (Figure 3). Samples from IVR had a slightly higher relative abundance (2.0–3.9%) of Rhodospirillaceae compared to those from PGR stations. Water column samples contained Rhodobacteraceae, Flavobacteriaceae and Rickettsiales-related families as the most common groups from both reefs. Burkholderiaceae were found only in low abundance (0.1–0.3%) in sediment and water column samples from IVR.

The three water column samples collected in different years during alternative seasons or from different reefs all shared the presence of Halomonadaceae (from 4.7% to 8.2%). In the dry season transition, members of Mamiellaceae were found in PGR samples but at higher abundance in the water column (13.5%) versus only 2.3% in sediments, and this low abundance trend was also reflected from IVR sediments (3.1%). In the same season, both reefs shared taxa belonging to the Rickettsiales (26% at IVR and 17.3% at PGR, respectively) as the most abundant order. In the rainy season, members of this order (20.9%) were also present at IVR in the water column, following the high relative abundance of Synechoccaceae (23.3%). 

Vibrionaceae were predominant in the microbiota of sediment from both reefs, although the two reefs exhibited markedly different microbial taxonomic composition. The three different sediment samples shared the presence of the Lentisphaeraceae family (ranged from 0.5% to 4.4%) (Table 4). The Vibrionaceae was present in all sediment samples, with the highest frequency in IVR sediments in the rainy season (10.2%), but with a low relative abundance in spring (1.7%). Although this family did not dominate PGR sediments, it made a substantial relative contribution (5.2%) to the total diversity. The PGR sediments were dominated by Flavobacteriaceae (15.8%). The three different sediment samples from the two reefs shared Lentisphaeraceae (ranging from 0.5% to 4.4%). The Bacillaceae, one of the eight families commonly found (29.4%) among the cultured isolates mix (IVR-CMix), was only rarely found (at 1.1%) in IVR sediments from the rainy season. Planococcaceae, well represented (2.5%) among the cultured isolates, were absent from the environmental samples. Pseudomonaceae members present among the isolates mix (31.1%) were also found in environmental samples from IVR in the rainy season, but in a very low relative abundance (1.6% from the water column and 0.3% from sediment). Exiguobacteraceae, in high relative abundance within the isolates mix (31.1%), were only detected in water column samples from IVR in the rainy season and only at low relative values (0.8%).

**Figure 4:**
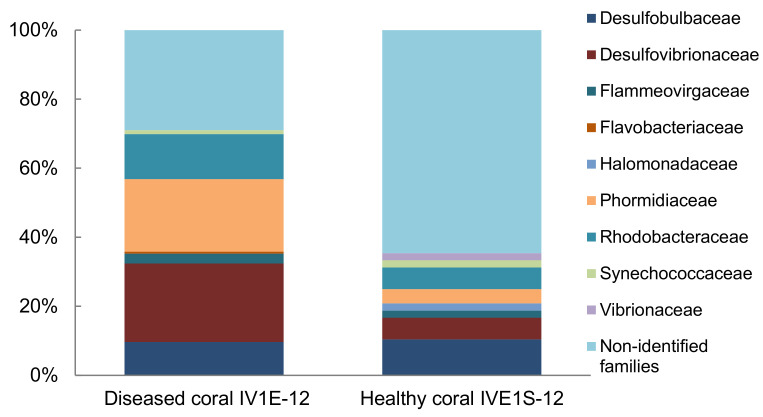
Relative composition (% total) of identified bacterial families represented by 16S rDNA sequences from apparently diseased (IV1E-12) and healthy coral (IVE1S-12) collected in mucus slurry from the surface of the massive starlet coral *Siderastrea siderea* at Isla Verde Reef (March 2012) in the Veracruz Reef System.

Comparison of the family-level bacterial community associated with apparently diseased (IV1E-12) versus healthy (IV1S-12) coral indicated a heavy dominance of Vibrionaceae but a lower proportion of Desulfovibrionanceae and Rhodobacteraceae in healthy relative to diseased coral (Figure 4). No linkage was evident with respect to pathogenicity of individual taxa towards coral host communities.

### 3.3. Diversity Analysis

**Figure 5:**
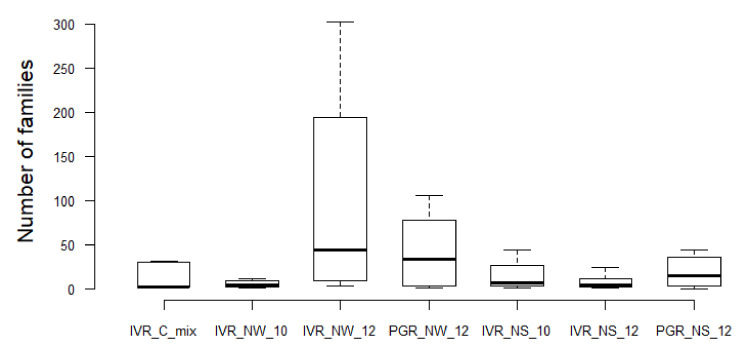
Boxplots illustrate the variation of bacterial families among samples from different geographical locations, seasons, and matrix types. The boxes show the 25–75 percentile values; the horizontal bars indicate the median, and the whiskers indicate the minimum and maximum data points. Mixed cultures (Isla Verde Reef) = IVR-C-Mix; Water column (IVR) = IVR-NW-10 (September 2010); IVR-NW-12 (March 2012); Water Column (Punta Gorda Reef) = PGR-NW-12 (May 2012); Natural sediment (Isla Verde Reef) = IVR-NS-10 (September 2010); IVR-NS-12 (March 2012); Natural sediment Punta Gorda Reef = PGR-NS-12 (May 2012).

The Shannon-Wiener diversity index (1) confirms the data presentation in Table 4 and Figure 3 that among geographical location and matrix types, the sediments of PGR in 2012 represented the highest biodiversity at the family level (H’ = 1.94), with the highest number of bacterial families (mostly from unidentified species), followed by the sediments of IVR 2010 (H’ = 1.90) (Figure 5). The IVR cultures mix was the least biodiverse (H’ = 1.37). The calculation of dominance with Pielou’s evenness index (2), indicated that the sediments of PGR 2012 displayed the most even bacterial community (J’ = 0.58), while the IVR cultures mix showed the least even community (J’ = 0.41). In all cases, the dominance tendencies were apparent but not overwhelming among families.

## 4. Discussion

The low success rate of isolation and growth of monoclonal bacterial isolates from diverse marine ecosystems has long been recognized as a critical limitation in valid comparisons of genetic diversity among populations and from different geographical locations [27]. Many bacterial genotypes that can be detected with molecular techniques in natural environmental samples are simply not susceptible to successful culture in the laboratory. Conversely, many taxa can be relatively overrepresented in culture because of favorably selective growth conditions. For example, although members of the Firmicutes were not abundantly represented in the environmental samples from the VRS, they comprised 65.5% among the successfully cultivated isolates. This is probably due to the selection for growth on LB culture medium commonly used for culturing the Firmicutes group.

Extrapolation of the responses of bacterial cultures to abiotic environmental factors (nutrients, temperature, salinity, etc.) to the natural environment has also proven difficult, particularly with respect to seasonal fluctuations. Most marine bacteria from coral reefs do not exhibit favorable growth when cultured in nutrient-rich media [28], particularly those from oligotrophic tropical oceanic environments. Nevertheless, in the current study, many bacterial isolates were successfully brought into stable culture when grown on highly nutrient-enriched LB medium. The highly enriched medium is expected to select for typically fast-growing bacteria under eutrophic conditions, and likely accounts for the fact that the species composition of the cultured isolates is glaringly distinct from that of the water column or sediment bacterial communities. This result is very similar to those for bacterial culture attempts from other marine environments, particularly from oligotrophic systems. 

Environmental data suggest that bacterial community composition of the VRS may not be strictly selected for oligotrophic conditions. Although low nutrient concentrations have been considered characteristic of the VRS, dissolved inorganic nitrogen (NO_3_ + NO_2_ + NH_4_) was shown to vary by approximately 50% and dissolved inorganic phosphate (PO_4_) by three-fold throughout the year [29]. During the rainy season, the nutrient status of the VRS may shift from rather oligotrophic to nutrient-enriched because of the nutrient flux from the rivers that run into the system [30]. In the Veracruz coastal zone, nitrate concentrations between 6.5–43.5 μM and inorganic phosphate concentrations between 4.2–20.0 μM have been recorded [31]. The seasonally high nutrient flux rates and low ambient N:P ratios suggest considerable P-loading to the coastal environment, including the VRS. This multi-year study, conducted between 2005 and 2012, revealed an average increase in nitrate concentration from 20.3 to 23.9 μM and of inorganic phosphate from 8.7–9.5 to 12.4 μM, indicating a progressive eutrophication of the system. 

The 16S rRNA coding gene has proven to be universal, conservative and to yield easy to align sequences for taxonomic identification of natural populations and isolated bacterioplankton and bacteriobenthos [28,32]. In the VRS study, molecular taxonomic information from the 16S rDNA gene was complemented by confirmatory analyses of biochemical characteristics, including enzymatic and anti-microbial activity, and morphological profiles of certain cultured isolates. Isolated bacteria from the VRS presented cell and colony size, color and morphology characteristic of these taxa as reported previously from other marine environments [28,32]. Consistent with most other marine isolates, such as *Oceanobacillus iheyensis* Lu et al. HTE831 [33], they showed predominantly the bacillus form, and were typically G(+), halotolerant, and aerobic. Notably, most of the isolates were metabolically active and produced a range of hydrolytic enzymes (lipases, esterases, chitinases, and amylases). Under these circumstances, it is likely that certain of these bacteria can actively degrade organic matter and affect the chemical ecology of their microenvironment. 

Halotolerant/halophilic bacteria capable of growth at higher salinities than normal for oceanic seawater were expected [34] because most marine prokaryotes are moderate halophiles [35]. Registered salinity at IVR was between 34–37 and this is consistent with the finding that all isolates, both thermophilic and mesophilic, were moderately halophilic. Thermophilic isolates able to grow at extreme NaCl concentrations (up to 2M) were those isolated from the bacteriobenthos (IVR-B-09-42, IVR-B-09-45, IVR-B-09-46), as well as several mesophilic strains (IVR-T-09-73, IVR-T-09-75, IVR-B-09-83). All of these halotolerant isolates were either associated with loose mucus biofilms from the reef or found adhered to seagrass. The mesophilic bacteria were observed to produce biofilms and precipitates as the concentration of NaCl was increased beyond 1 M. This assay was another differentiation criterion among the isolated strains that showed close phenotypic similarities [36]. Due to the variation of environmental and nutritional conditions, these strains are likely able to resist higher salt concentrations. Variations in temperature, culture media and pH may expand the tolerance window from slight to extreme halophily in marine strains, such as those of *Oceanobacillus iheyensis* [33]. Further details on the growth and morphological characteristics are not included here because the primary focus was on comparative diversity and classification of natural populations versus culture-selected strains.

Effects on bacterial community composition via seasonal shifts in temperature, salinity, pH and advective currents, are a well-known phenomenon. In general, there were no apparent seasonal differences on bacterial community structure in the water column at IVR between the rainy season (e.g., September 2010) and a transitional period between the northerly winds and the dry season; samples from PGR collected in the dry season (May 2012) also exhibited roughly similar bacterioplankton composition. Furthermore, there was a consistent pattern in bacterial composition from the sediments at both reefs, with α- and γ-proteobacteria dominating at both reefs during the three seasonal comparisons, but at the family level there were significant differences in abundances within the sediments between IVR and PGR.

Environmental evidence suggests that terrestrial run-off may be a more important seasonal determinant of bacterial diversity and distribution in the VRS than seasonal water temperature shifts alone. Variability of the water temperature and salinity in the VRS is mainly determined by the discharges of the Jamapa and Papaloapan rivers. During the rainy season, the Jamapa River discharges up to 180 m^3^ s^−1^, whereas this river is, in general, characterized by weak discharge (1.9 × 10^6^ m^3^ year^−1^) during most of the year [9,10]. The Papaloapan River, situated ca. 80 km southeast from the VRS, has an annual discharge of ca. 36 × 10^6^ m^3^ year^−1^ [10]. Based on river discharges and wind velocity, three seasons were distinguished: (1) the northerly winds period (March to June), at least 80% of the time with wind intensities >5 m s^−1^; (2) the rainy season (June to October), when river discharges increase to >30 m^3^ s^−1^, with low wind intensities (approximately 50%) alternating between southerlies and northerlies; and (3) the dry season (October to March), with total river discharges < 30 m^3^ s^−1^, and when northerlies blow at least 70% of the time at velocities < 5 m s^−1^. During March there are variable transitional conditions, typically lasting up to 20 days [10,37]. This seasonal classification is in accordance with the combined variations in water temperature, salinity and density [37].

In principle, seasonal shifts in the aquatic regime could account for differences in bacterial composition and abundance, but these effects on diversity were not evident. Definitive seasonal variations are not possible to derive from these diversity data because of the lack of multi-year comparisons from various habitats and matrices. Limited environmental data from the water column (temperature, salinity, O_2_, pH) collected during sampling (Table 1) are not conclusive but do reflect typical conditions expected for this time of year in the VRS. For example, in March 2012, the temperature characteristics were those represented by the dominant northerly winds period, unlike in March 2011, the beginning of the dry season. It is important therefore to consider the atmospheric variables of each year to determine the seasonal variability [10,37]. A multi-decadal (30-year) comparison of average seasonal meteorological data from the Gulf of Mexico (air temperature, wind velocity, precipitation) [38] indicates no major anomalies from expected conditions during the yearly periods of bacterial sampling of the VRS, except perhaps for the transitional period in March. In any case, seasonal shifts in prevailing meteorological conditions are expected to be only indirect drivers of bacterial diversity and abundance, with the exception of major oceanic disturbances caused by hurricanes and storms, which are frequent in the region. 

Compositional shifts of bacterial communities (but not abundance) have been shown to be due to pH changes, particularly affecting some pH-susceptible groups, such as γ-proteobacteria*,* Flavobacteriaceae, Rhodobacteraceae, Campylobacteraceae and other less abundant taxa [39]. In the case of the VRS, the consistent pH (8.0–8.2) of the water column during the four seasonal sampling periods provides no evidence for acidification affecting the water column communities. The inter-seasonally consistent pH at both reefs is reflected in the similar abundances of α- and γ-proteobacteria and sub-class Synechococcophycideae, order Rickettsiales, and Synechococcaceae and Rhodobacteraceae at the family level.

Among selective environmental factors, salinity has been reported to have the strongest impact at phylogenetic levels; with a rise of salinity α- and γ-proteobacteria can increase, contrasting with a decrease of β-proteobacteria with a decrease of salinity [40]. Bacterial samples from the VRS contained mainly α- and γ-proteobacteria, and while PGR did not reveal any β-proteobacteria, they were found at IVR but typically at <0.7%, with only one exception which contained substantial β-proteobacteria (8.5%) from the water column sampled in the fall (IVR-NW-10).

Community composition of marine bacteria can respond to local environmental conditions, such as salinity and temperature effects on biofilms [41], but there was little to distinguish the ambient regime between the reefs. Temperature and salinity ranges were similar, whereas pH was constant (around 8.2) and dissolved oxygen showed only slight variations between the two reefs. In fact, within a season and given ecological compartment (water column, attached to substrate, sediments), the bacterial community composition was similar at the reefs. Specifically, the water column exhibited almost the same bacterial family composition during 2010 and 2012 at both reefs (IVR-NW-10, IVR-NW-12, PGR-NW-12) (Table 4). Furthermore, the sediments showed similar relative bacterial composition at both reefs and between seasons, albeit with different abundances. 

### 4.1. Growth and Phylogeny of Cultured Isolates

Bacteria from surface waters were the focus of our studies on cultured isolates because these populations are expected to be subject to most rapid environmental changes [42]. This led to the hypothesis that they may yield more metabolic and enzymatic adaptations capable of adjustment to variable environmental conditions, and hence useful and novel features to exploit for biotechnological purposes. The mesophilic cultured isolates derived from the VRS represent well-described taxa known from coastal marine environments around the world [43,44,45]. Gram-positive (G+) bacteria, such as *Jeotgalicoccus halotolerans* Yoon et al. and *J. psychrophilus* Yoon et al. representing more extreme environments, have been isolated from Korean fermented marine products (jeotgal fish sauce) [45]. The γ-proteobacteria, such as those of genus *Pseudomonas*, are frequently isolated from coastal waters [43,44]. Heat-resistant strains of *Exiguobacterium* have been previously reported [46], but in our study, the isolate of this genus was unable to grow above 50 °C, unlike other mesophilic isolates.

The presence of thermophilic microorganisms in coral reefs is significant because these nominally mesophilic environments can reach maximum temperatures even exceeding 30 °C. On the other hand, cultivable thermophilic microorganisms, specifically Archaea, have been reported in non-thermophilic coastal zones, including superficial waters of the Pacific Ocean, temperate waters in the North Atlantic, the Mediterranean and even the Antarctic [47]. 

In Mexico, the VRS is influenced by the contribution of rivers from hydrothermal zones, like Río Pescados that crosses the hydrothermal system El Carrizal, and is associated with the Pico de Orizaba volcanic system at 120 km from the port of Veracruz. Hence at least some of these bacteria found within the VRS could have a remote origin, probably from submarine hydrothermal upwellings, and the bacterioplankton could have been dispersed by marine currents. 

Most thermophilic isolates from the VRS belonged to the genera *Geobacillus* and *Aeribacillus* which have been described as aerobic or facultative anaerobic neutrophilic bacilli [48]. These are common inhabitants of geothermal environments and found in soil, water, contaminated food, and oceanic sediments, with permissive normal growth at temperatures between 30–75 °C [48,49] and an optimum between 60–80 °C [50]. Five phyla from the VRS have a match below or equal to 98%, but the thermophilic strains IVR-B-09-44, IVR-T-12-21, IVR-S-10-7, the mesophilic IVR-T-09-74, IVR-B-09-83 and the psychrotolerant IVR-W-10-51 could belong to new phyla. 

Despite the wide distribution and abundance of proteobacteria and cyanobacteria in the marine environment, neither Pelagibacterales (SAR11), including *Pelagibacter ubique* Rappé et al. [51,52], nor the common cyanobacteria *Prochlorococcus* Chisholm et al. or *Synechococcus* Nägeli [28,51,53] were found among the isolates. This may be because *P. ubique* typically grows under oligotrophic conditions, whereas growth attempts with isolates in the laboratory were conducted upon highly nutrient-enriched LB media. Constraints on the culture of cyanobacteria such as *Prochlorococcus* are likely since experiments were not conducted under the necessary growth conditions for such photosynthetic organisms.

In this study, both mesophilic and thermophilic bacterial isolates were associated mainly to Firmicutes and Proteobacteria, and clustered into the classes Bacilli, and β- and γ-proteobacteria (Table 4). Although most isolated marine bacteria belong to the Firmicutes, a thermophilic strain IVR-T-12-23 was classified as a β-proteobacterium and two strains (IVR-S10-1 and IVR-W-10-51) belong to the γ-proteobacteria. These latter strains could be considered mesophilic or least facultative psychrotrophs [34,50] because they were isolated at 1 °C but grew better at 33 °C with agitation. Isolate IVR-T-12-23 originating from seagrass is phylogenetically most closely related to *Delftia acidovorans* (den Dooren de Jong) Wen et al. but with a poor match (85% similarity). Furthermore, *D. acidovorans* has been reported as a G(−) bacillus [54], whereas strain IVR-T-12-23 is G(+). The relatively low similarity at the genus level (97%) was insufficient to classify this strain even within *Delftia* Wen et al. 

The microbiome of coral surface mucus includes bacteria that may comprise an important component of the microbial food web. The bacterial component exhibits a dynamic relationship with overall microbial communities of coral [12,55], contributing to both the diversity and distribution of species-specific associations, as identified on the mucus of coral species from Brazil [56]. Heterotrophic bacteria located on the surface of corals, as contrasted with the less common photoautotrophic cyanobacteria, grow primarily by organic nutrient assimilation from the mucus layer. The contribution to the microbial loop is initiated after bacterial consumption by microflagellates, these latter by ciliates and finally by copepods and other Metazoa associated with coral reefs [55]. 

Proteobacteria was the dominant phylum represented in environmental samples from the water column and sediments at both reefs of the VRS. According to previous metagenomic studies, Proteobacteria tend to dominate the bacterioplankton and sediments of reef habitats, including deep cold-water coral reef systems in Norway [57]. In the present study of the warm-water VRS system, phylum Firmicutes was dominant, followed by Proteobacteria in second rank, as represented among the cultivable isolates; this is consistent with the fact that many of the known cultivable marine bacteria belong to Firmicutes. Note that this comparison is restricted to cultures from IVR; no cultures were available from PGR. Studies on cultured isolates from marine sediments from the Mediterranean Sea indicated that Firmicutes and Actinobacteria were dominant, with *Bacillus* as the most frequent genus [58]. Findings from marine sediments of the South Atlantic also cite Firmicutes, Actinobacteria and γ-Proteobacteria as the dominant cultivable bacteria [59].

Members of the genus *Geobacillus* are commonly found in geothermal environments and are widely distributed in soil, hot springs, contaminated food and ocean sediments [48,49]. With high thermal tolerance, *Geobacillus* isolates are typically able to grow at 30–75 °C [48,49] and optimally at 60–80 °C [50]. The strains IVR-B-09-44 and IVR-B-09-45 in the current study are aerobic bacilli, neutrophilic and catalase positive as consistent with *Geobacillus* and were first assigned to *G. pallidus* (Scholz et al.) Banat et al. on the basis of 16S sequences; in the final analysis, the strains were reclassified as *Aeribacillus* sp. [48] with a description as an aerobic bacillus, alkaline-tolerant and positive to catalase screening.

*Lysinibacillus* Ahmed et al. is a G(+) bacterium found globally in both soil samples and fish tissues [60]. The thermophilic strains IVR-W-10-57 and IVR-S-10-7 from the VRS are similar to *Lysinibacillus* and marine Pseudomonaceae, respectively.

The β-proteobacteria are common in coastal ecosystems, with chemolithotrophic degradative potential; this group also includes some higher animal pathogens such as *Neisseria* Trevisan and *Burkholderia* Yabuuchi et al. Although most β-proteobacteria have been reported as mesophilic, there are thermophilic strains (most of them chemolithotrophic), such as *Thiomonas thermosulfata* Moreira et Amils*, Thermothrix azorensis* Odintsova et al.*, T. thiopara* Caldwell et al.*, Tepidimonas ignava* Moreira et al.*, Caldimonas manganoxidans* Takeda et al.*, Tepidiphilus margaritifer* Manaia et al., and *Hydrogenophilus hirschii* Hayashi et al. isolated from hot springs at Yellowstone Park in western USA [61]. These thermophilic strains grow optimally around 65 °C [61], as did our strains from the VRS. All these reported thermophiles, except for *T. margaritifer*, originate from hydrothermal systems, an environment unlike that of the origin of our isolates from the biofilm covering seagrass.

There are reports of thermophilic bacteria, namely the anaerobic *Kosmotoga arenicorallina* Nunoura et al. and *Sulfurivirga caldicuralii* Takai et al., flourishing around a coral reef in the south of Japan; this locality, however, is associated with a shallow marine hydrothermal system [62,63]. The presence of thermophilic strains of marine *Bacillus* from hydrothermal vents has been detected years ago [64], but they are now known to be closely related phylogenetically to *Geobacillus*, the homologous genus. In the Gulf of California near Guaymas, Mexico, Teske et al. [65] found populations of this genus close to geothermal habitats in marine sediments. This could also explain the presence of *Geobacillus* in the VRS, since this system receives freshwater inflow from the Pescados River associated with the hot spring El Carrizal. 

Most Firmicutes are G(+) and Proteobacteria are G(−), coinciding with VRS strains, except for the proteobacterium IVR-T-12-23 that stained G(+), and a few other Firmicutes that were G(−). These isolates are possible new species but are not strains known to originate directly from human or soil sources.

### 4.2. Molecular Phylogenetics and Diversity of Associated Coral Reef Bacteria from Natural Populations

Coral reefs are well-described marine environments, where healthy microbial community dynamics often characterized by low nutrient concentrations but high recycling and regeneration rates. Oligotrophic systems such as coral reefs are suggested to have a relatively low microbial diversity [53]. On the contrary, Venter et al. [66] found a wide variety of microorganisms in the oligotrophic Sargasso Sea—1800 bacterial species including 148 new bacterial phylotypes. Similarly, in the studied ecological compartments of the VRS, here we report the presence of a total of 223 families, which implies high microbial diversity at this taxonomic level. On the other hand, at the “species” level, only about 8000 distinct amplicons were identified—this is considered low for most healthy marine environments. This implies either that the VRS is not a typical oligotrophic coral reef system or that oligotrophic status is not a valid indicator of low microbial diversity. 

All the sequences from the VRS were classified into 33 phyla, yielding the Proteobacteria cluster as dominant in all environmental samples. Similar results have been reported for large oceanic areas, where the Proteobacteria clade (SAR11) represented up to 35% of the prokaryotic picoplankton diversity in surface water, with highest species abundance in the North Atlantic [67].

The studied reef system is adjacent to the Atlantic Ocean, from where the reef-associated water masses are derived [68]. Advective transport can explain the dominance of Proteobacteria, amplified by the effects of microscale conditions within the VRS. The Proteobacteria clade has been reported as the most taxonomically rich group in reef zones, comprising up to 68% of coral holobionts [55]. Conversely, as in our VRS study, cyanobacterial taxa are typically scarce within reefs, as well as in sediment samples from these systems. This is perhaps because, in general, they are free-living in the water column and relatively independent of organic nutrients associated with sediments or organic-rich benthic substrates. 

Firmicutes has been reported as a characteristic group in some coral holobionts [69], but this clade was uncommon in the environmental samples from the VRS. Yet the isolate mix sample from the VRS contained Firmicutes as the dominant group (maximum 65.5%) followed by Proteobacteria (maximum 38%); similar dominances were reported from marine sediment isolates from the Mediterranean Sea and the South Atlantic [59]. In fact, this phylum was abundantly represented in the nutrient-rich LB cultures, indicating that they may be favored in high nutrient environments. Within coral reefs, most bacteria associated with mucus are aerobic and heterotrophic, like some members of the phylum Firmicutes, found living at the mucus surface of corals rich in polysaccharides [12].

After Proteobacteria, the water column of the world oceans tends to be dominated by members of the phylum Cyanobacteria, with high abundances of photosynthetic coccoid picoplankton such as *Prochlorococcus* and *Synechococcus* [28,51,53]. At the class level, within the VRS, however, Synechococcaceae followed Pseudomonadaceae in taxonomic diversity within the water column.

At the family level, the diversity dominance patterns differed between seasons, even if the sequences were derived from the same reef, and within the reef among habitats. At IVR, the Synechococcaceae (23.3%) was the main group in the water column in September, whereas dominance shifted to unclassified Rickettsiales (26%) in March. In contrast, in sediments, the samples taken in September were dominated by Vibrionaceae (10.2%) and those from March by Alteromonadaceae (9%). PGR was sampled only in the dry season, with unclassified Rickettsiales as the most abundant group in the water column (17%), but with higher representation by Flavobacteriaceae (15.8%) in sediments.

In general, at the genus level within the VRS, *Prochlorococcus* was more abundant than *Synechococcus*. This is consistent with previous findings, where *Prochlorococcus* was dominant in the tropical Atlantic. At the same time, α-proteobacteria (SAR11) have also been reported as a dominant group in surface waters [67], in agreement with our VRS study where it was the most abundant class represented in the waters column from both reefs. There is a high percentage of Cyanobacteria in the reef population structure benthic holobionts [2]. 

The Bacteroidetes was the third most abundant group represented in the water column and sediment samples of both reefs. This bacterial contingent could be related to organic matter processing, particularly in association with known eukaryotic harmful algal blooms in the study area [31,70], but this would depend on the magnitude of the bloom events and subsequent release of organic matter during bloom senescence. 

The Flavobacteriaceae and Halomonadaceae are considered to be moderately halophilic [71]; both mesophilic and thermophilic isolates grow at moderate halophilic conditions. The former group was found in the water column and the latter in sediments of the VRS, and this reef system could therefore be an important source of halophilic organisms. 

Most of the halotolerant bacterial isolates from the VRS belong to Firmicutes and Bacteroidetes. Although none of the isolated clades were detected in environmental samples, and vice versa, this does not necessarily mean that they are absent. Pyrosequencing studies of bacteria from hypersaline water report Firmicutes abundances of 15% in systems with a salinity gradient up to 6.4% [72].

Members of the phylum Actinobacteria have been previously isolated from the marine environment [59]; this phylum was poorly represented, however, (0.2% at IVR and only 0.1% at PGR) in sediment samples in the VRS. Meanwhile, SAR406 or Marine Group A, characteristic of oceanic water [67,73] was found in all environmental samples, but in low quantities (0.4 to 3.1%). The two dominant phyla, Firmicutes and Proteobacteria, represented among the cultured isolates were also encountered in the environmental samples, although Firmicutes only in a very low relative abundance (typically < 1%) and only once in a sediment sample (3.2%) collected from IVR (M7). 

Worldwide there are more than 20 reported coral diseases, but only six isolated agents responsible for them have been identified [16]. Most coral pathogenic agents (*Vibrio* Pacini, *Thalassomonas* Macián et al. or *Serratia* Bizio spp.) belong to the γ-Proteobacteria [16], also a highly represented class in sediment samples from the VRS (19.2–35.7%). There are reports related to the distribution and prevalence of diseased corals inside the VRS [8,74]. Horta-Puga and Tello-Musi [75] found that 4.2% of the corals in the VRS were diseased, and dark spot disease (DSD) was highly prevalent (41.9%) in the VRS. A pronounced increase in coral disease incidences was registered from 2006 to 2010, with especially high values at IVR [76].

There is no published information on the interactive role of microorganisms and environmental conditions regarding the health status of corals in the VRS. Furthermore, there are no previous taxonomic or phylogenetic studies about the normal microflora found in this system, even when the close relationship among certain microorganisms and coral diseases has been established, such as for bleaching (*Vibrio shiloi* Kushmaro et al.), bleaching and lysis (*V. coralliilyticus* Ben-Haim et al.), aspergillosis (*Aspergillus sydowii* (Bainier et Sartory) Thom et Church), white band disease (*V. carchariae* Grimes et al.), white plague disease (*Thalassomonas loyana* Thompson et al.), white pox disease (*Serratia marcescens* Bizio), yellow band disease (*V. alginolyticus* [Miyamoto et al.] Sakazaki) and black band disease, apparently associated with a microorganismal consortium [16].

The health status of the corals from which the mucus bacteria were collected in the current study was not determined; apparently diseased specimens were identified only by the presence of visually remarkable necrotic discolorations—characteristic of black spot disease (BSD) (alternatively called black spot syndrome; BSS) common in the Caribbean [77] but of uncertain etiology and transmissibility [78]. In a study of bacterial association with the coral *Siderastrea sidereal* [79], the genera *Oscillatoria* Vaucher ex Gomont and *Vibrio* were present in the bacterial community of BSD-afflicted specimens, but not in healthy ones; cyanobacteria were dominant in diseased corals (63.28% of the bacterial community), whereas healthy corals contained 52.16% Proteobacteria. The profile distinction concurs with the finding shown in the current study (Figure 4) that associated bacteria can be diagnostic indicators for coral disease and reef health status. Detailed studies on the interactions between potentially pathogenic bacteria and the seasonality and health status of affected corals in the VRS are required to resolve this issue. 

The VRS sediments may host a *Vibrio* pathogen because Vibrionaceae are commonly present (3.8–10.2%) in the sediments of this study. Nevertheless, even though potential coral pathogenic species may be present in both reefs, it does not necessarily mean that the corals are infected with such pathogens, because bacteria that live in/on the coral holobiont are not the same strains as those of the surrounding water [2,69]. Furthermore, if the holobiont is in healthy equilibrium with its natural bacterial complement it will normally resist disease.

Although both reefs are close to the coast and near sewage discharge sites for the city, we did not find any member of the Enterobacteriales, as would be characteristic of fecal contamination. The current and wind patterns, among other factors, have an important role in bacterial dispersion and likely account for the absence of coliforms in the IVR and PGR samples.

The earliest publications on marine microbial populations report a clear diversity difference between bacterioplankton and bacteriobenthos recovered from the water column and sediment samples, respectively [80]. For example, within the VRS, Spirochaetes were found only in the IVR sediment sample (3.3%) collected in the rainy season. In descending taxonomic rank and reaching family level, the difference between the microbial diversity and abundance in the water and sediment samples becomes even more evident. At this level, microbial taxonomic diversity was higher in the sediment samples compared to those from the water column, and much higher than among the cultured isolates.

The bacterial communities in VRS sediments and the water column were distinct, even if they belonged to the same reef, but very similar between reefs if they originated from the same habitat. The fact that the two studied reefs belong to the same subzone within the park [68] perhaps accounts for the close similarity between their bacterial communities found in both the surrounding water and sediments. Previous studies reported that microbial communities appear to be specific to certain corals in different locations; in some cases, however, they can differ because of the influence of different stressors [2,81]. The close proximity of the reefs to each other and to the port of Veracruz, a potential source of high anthropogenic stress, tends to indicate that environmental stressors to their respective ecosystems are similar. Furthermore, the low contingent of pathogenic bacteria, either of anthropogenic origin from outside the VRS or generated endogenously from diseased coral, is a positive sign that these reefs are in a tolerable equilibrium, at least with respect to their microbial components. 

The dominant taxonomic groups (Proteobacteria, Cyanobacteria and Bacteroidetes) in the water column and sediments of both studied reefs of the VRS were consistent with the groups previously found to be dominant in marine coastal environments in the northeastern Gulf of Mexico [82]. In this context, sand beaches polluted by the Deepwater Horizon oil spill in 2010 in the northern Gulf of Mexico are highly populated by γ-Proteobacteria, including oil degrading bacteria [83].

The dominance by γ-proteobacteria in the bacteriobenthos in the studied sediment samples of the VRS could also be related to historically low levels of oil pollution transported to the reefs, but we did not explore the degradative capacity of γ-proteobacterial isolates nor determine the spectrum and concentration of exogenous hydrocarbons within the VRS. Such screening studies of both the water column and sediments of reef systems in the Gulf of Mexico would be required to demonstrate hydrocarbon degrading capabilities of the bacterial community, as well as to establish relevant shifts in the biodiversity profile as a consequence of recent or historical exposure to oil contamination. 

## 5. Conclusions

Many common sequences of 16S rRNA were found between cultured isolates and those recovered directly from environmental samples from the VRS, particularly among the Firmicutes and Proteobacteria. Nevertheless, the many distinct sequences revealed deep differences between abundances among isolates and environmental samples at lower taxonomic levels (family, genus and species). In any case, the lower diversity of the cultured isolates compared with natural bacterial communities reflects the well-known “genetics of survivors” limitation due to the failure to provide a broad spectrum of permissible growth conditions in culture. 

The bacterial composition of sediments is more diverse than found in the water column. The diversity variations observed between the water column and sediments can be attributed to the different conditions that exist in these habitats, nutrients, exposure to wind-driven mixing and tidal currents, and the presence or absence of suitable substrates for adhesion. Most significantly, in spite of the expected high impact of anthropogenic stressors on the VRS, there is no discernable evidence from the diversity patterns of either cascading disequilibrium in the composition of the microbial contingent or pathogenic loading to indicate an unhealthy reef community. The bacterial communities within the water column and sediments are rather diverse and represent a generally balanced cell abundance and taxonomic representation in their respective compartments. This tends to indicate that reef response to seasonal and anthropogenic influences at least at the studied sites of the VRS is rather robust and resilient. The stability and compositional diversity of bacterial communities as a reflection of coral reef health should always be considered as a critical element for management decisions for environmental policies.

## Figures and Tables

**Table 1:** Cultured bacterial isolates from the Veracruz Reef System indicating geographical location and ambient environmental parameters of field sampling for isolation. IVR = Isla Verde Reef; PGR = Punta Gorda Reef; T = *Thalassia testudinum*; B = uncharacterized biofilm; W = water column; S = substrate; N = natural (non-cultured). Other designations are laboratory codes for field samples or cultures.

Code	Geographical Origin	Ecological Compartment	Date of Collection	Ambient Environmental Parameters				
				Depth (m)	T °C	Salinity	O_2_ (mg·L^−1^)	pH
IVR-T-09-31	Isla Verde Reef	*Thalassia testudinum* (seagrass)	August 2009	1–1.5	29.6	36.0	6.65	8

IVR-T-09-32								
IVR-T-09-72								
IVR-T-09-73								
IVR-T-09-74								
IVR-T-09-75								
IVR-B-09-42		Biofilm						
IVR-B-09-43								
IVR-B-09-44								
IVR-B-09-45								
IVR-B-09-46								
IVR-B-09-81								
IVR-B-09-83								
IVR-W-09-61		Water column						
IVR-S-09-52		Sediments						
IVR-W-10-2	Isla Verde Reef	Water column	September 2010	10–11	27.0	34.0	ND	8.2
IVR-W-10-57								
IVR-W-10-51								
IVR-S-10-7		Sediments						
IVR-S-10-1								
IVR-NW-10		Water column						
IVR-NS-10		Sediments						
IVR-T-12-21	Isla Verde Reef	*Thalassia testudinum* (seagrass)	March 2012	10–11	28.0	36.6	3.6	ND
IVR-T-12-22								
IVR-T-12-23								
IVR-S-12-91		Sediments						
IVR-S-12-92								
IVR-NW-12		Water column						
IVR-NS-12		Sediments						
IV1E-12		Diseased coral						
IVE1S-12		Healthy coral						
PGR-NW-12	Punta Gorda Reef	Water column	May 2012	3.4	26.0	37	4.6	ND
PGR-NS-12		Sediments						

**Table 2:** Taxonomic assignments of isolated cultured thermophilic bacteria from the Veracruz Reef System. Only species identities >75% are indicated. IVR: Isla Verde Reef; BLAST: Basic Local Alignment Tool; RDP: Ribosomal Database Project.

	BLAST		RDP	
Isolate	% Similarity to Known Species or Genera	Species	% Similarity (Phylum & Species)	Phylum: Species
IVR-B-09-44	99	* Aeribacillus * * pallidus *	80	Firmicutes
IVR-B-09-45	100	* Aeribacillus pallidus *	100 & 91	Firmicutes: *Aeribacillus pallidus*
IVR-T-12-23	93	* Delftia * sp.	100 & 85	β-Proteobacteria: *Delftia acidovorans*
IVR-T-09-31	89	* Geobacillus * sp.	100	Firmicutes
IVR-T-09-32	94	* Geobacillus * sp.	100	Firmicutes
IVR-B-09-42	97	* Geobacillus thermodenitrificans *	100 & 88	Firmicutes: *Geobacillus thermodenitrificans*
IVR-B-09-43	88	* Geobacillus * sp.	100 & 89	Firmicutes: *Aeribacillus pallidus*
IVR-B-09-46	88	* Geobacillus * sp.	100 & 89	Firmicutes: *Aeribacillus pallidus*
IVR-W-10-2	94	* Geobacillus thermodenitrificans *	100 & 94	Firmicutes: *Geobacillus thermodenitrificans*
IVR-T-12-22	95	* Geobacillus * sp.	98	Firmicutes
IVR-S-12-91	94	* Geobacillus thermoglucosidasius *	100 & 89	Firmicutes: *Geobacillus toebii*
IVR-S-12-92	92	* Geobacillus * sp.	100 & 86	Firmicutes: *Geobacillus thermoglucosidasius*
IVR-W-10-57	95	* Lysinibacillus * sp.	99 & 85	Firmicutes: *Geobacillus toebii*
IVR-S-10-7	79	Undefined marin Pseudomonaceae sp.	100	Firmicutes

**Table 3:** Taxonomic assignments based on 16S rDNA sequencing of isolated cultured mesophilic bacteria from Isla Verde Reef (IVR) within the Veracruz Reef System. BLAST: Basic Local Alignment Tool; RDP: Ribosomal Database Project.

	BLAST		RDP	
Isolate	% Similarity to Known Species or Genera	Species	% Similarity (Phylum & Species)	Phylum: Species
IVR-T-09-74			94	Firmicutes
IVR-S-09-52	98	*Bacillus* sp.	99 & 99	Firmicutes: *Bacillus cereus*
IVR-B-09-81	99	*Bacillus cereus*	100 & 100	Firmicutes: *Bacillus* sp.
IVR-B-09-83			95	Firmicutes
IVR-T-09-73	99	*Bacillus pumilus*	100 & 96	Firmicutes: *Salirhabdus pumilus*
IVR-T-09-75	98	*Bacillus pumilus*	99 & 93	Firmicutes: *Bacillus pumilus*
IVR-T-09-72	99	*Exiguobacterium* sp.	100 & 96	Firmicutes: *Exiguobacterium aestuarii*
IVR-W-09-61	91	*Jeotgalicoccus* sp.	99	Firmicutes
IVR-W-10-51	94	*Pseudomonas* sp.	95 & 69	γ-Proteobacteria: *Serpens syringae*
IVR-S-10-1	97	*Pseudomonas fluorescens*	100 & 92	γ-Proteobacteria: *Pseudomonas fluorescens*

**Table 4:** Taxonomic distribution (% total) of bacterial populations from the water column and sediments compared with cultured isolates from Isla Verde reef (IVR), classified by pyrosequencing of 16S rRNA. Only specified groups representing ˃ ca. 3% of the total bacterial assemblage are included. Mixed cultures (Isla Verde Reef) = IVR-C-Mix; Water column (IVR) = IVR-NW-10 (September 2010); IVR-NW-12 (March 2012); Water Column (Punta Gorda Reef) = PGR-NW-12 (May 2012); Natural sediment (Isla Verde Reef) = IVR-NS-10 (September 2010); IVR-NS-12 (March 2012); Natural sediment Punta Gorda Reef = PGR-NS-12 (May 2012).

	Isla Verde Reef (IVR)			Punta Gorda Reef (PGR)	Isla Verde Reef (IVR)		Punta Gorda Reef (PGR)
	IVR-C-Mix	Water Column IVR-NW-10	Water Column IVR-NW-12	Water Column PGR-NW-12	Sediment IVR-NS-10	Sediment IVR-NS-12	Sediment PGR-NS-12
**Phyla (33)**	**3**	**8**	**12**	**14**	**27**	**18**	**26**
Firmicutes	65.5						
Proteobacteria	32.8	62.8	56.3	45.8	58.2	57.6	41.7
Cyanobacteria		23.3	21.6	25.6			8.6
Lentisphaerae					6.8	6.8	
Bacteroidetes		5.4	8.3	9.3	3.6	2.9	19.4
Other phyla	1.7	0.8	7.9	14.1	12.5	21.5	18.7
**Class or subclass (74)**	**3**	**11**	**24**	**19**	**50**	**29**	**48**
Bacilli	63.9						
α-Proteobacteria	32.6	39.5	40.7	30.5	35.7	24.9	19.2
Clostridia	1.7						
Synechococcophycideae		23.3	15.4	10.8			
γ-Proteobacteria		8.5	13.0	13.9	10.3	22.2	18.0
Lentisphaerae					10.8		
δ-Proteobacteria						4.4	
Flavobacteria							17.1
**Order (131)**	**5**	**19**	**46**	**33**	**82**	**44**	**75**
Bacillales	32.8						
Exiguobacterales	31.1						
Pseudomonales	31.1						
Synechococcales		23.3	15.4				
Rickettsiales		20.9	26.0	11.3			11.3
Rhodobacterales		12.4		11.2		8.0	
Alteromonadales					11.3	11.7	
Vibrionales					10.2		5.2
Oceanospirillales			11.3	11.3	5.4		
Lentisphaerales						5.4	
Flavobacteriales							16.1
**Families (223)**	**8**	**26**	**62**	**38**	**119**	**52**	**98**
Pseudomonadaceae	31.1						
Exiguobacteraceae	31.1						
Bacillaceae	29.4						
Synechococcaceae		23.3	15.4				
Other than Rickettsiales *		20.9	26.0	17.3			11.3
Rhodobacteraceae		12.4	9.0	11.2		8.0	
Vibrionaceae					10.2		5.2
Alteromonadaceae					8.0	9.0	
Lentisphaeraceae					4.4	3.4	
Undefined α-proteobacteria						5.1	
Flavobacteriaceae							15.8
Mamiellaceae				13.6			

* indicates undefined families than do not belong to Rickettsiales but are most similar to this order.

## Data Availability

Raw high-throughput sequencing reads were deposited in the National Center for Biotechnology Information (NCBI) Sequence Read Archive (SRA) GenBank database (experiment accession numbers MN103868—MN103887; BioProject submission ID numbers: SUB5868169; SUB5868184).
